# Impact of prior solid tumor on outcomes of hematopoietic stem cell transplantation for hematologic malignancies: a propensity score-matched study

**DOI:** 10.3389/fimmu.2026.1727469

**Published:** 2026-02-05

**Authors:** Jieya Luo, Mingyang Wang, Yunxia Zhou, Wenhao Wang, Wenbin Cao, Rongli Zhang, Xin Chen, Qiaoling Ma, Jialin Wei, Weihua Zhai, Yi He, Donglin Yang, Aiming Pang, Sizhou Feng, Mingzhe Han, Erlie Jiang

**Affiliations:** 1 State Key Laboratory of Experimental Hematology, National Clinical Research Center for Blood Diseases, Haihe Laboratory of Cell Ecosystem, Institute of Hematology & Blood Diseases Hospital, Chinese Academy of Medical Sciences & Peking Union Medical College, Tianjin, China; 2 Tianjin Institutes of Health Science, Tianjin, China; 3 Department of Hematology, Air Force Medical Center, Fourth Military Medical University, Beijing, China; 4 Haihe Laboratory of Cell Ecosystem, Tianjin Medical University, Tianjin, China

**Keywords:** hematopoietic cell transplantation-comorbidity index, hematopoietic stem cell transplantation, solid tumor, therapy-related hematologic neoplasms, transplant outcomes

## Abstract

**Background:**

The Hematopoietic Cell Transplantation-Specific Comorbidity Index (HCT-CI) assigns a high-risk score to patients who develop secondary hematologic malignancies following solid tumors, indicating an increased risk of non-relapse mortality (NRM). This study aimed to evaluate the impact of prior solid tumors on outcomes after hematopoietic stem cell transplantation (HSCT).

**Methods:**

From a cohort of 2,382 patients who underwent HSCT for acute lymphoblastic leukemia (ALL), acute myeloid leukemia (AML), or myelodysplastic syndrome (MDS) between January 2014 and July 2024, we included 43 (1.8%) with a history of prior solid tumors and 82 matched controls for analysis by 1:2 propensity score matching.

**Results:**

The solid tumor cohort predominantly comprised breast cancer (48.8%). With a median follow-up of 31.0 months, only one patient exhibited post-transplant relapse or metastasis of the solid tumor. Compared to the control group, patients with solid tumors exhibited higher ECOG scores (≥ 2: 23.1% vs. 9.5%, P = 0.049), lower platelet counts (35.5 vs. 72×10^9^/L, P = 0.010), a higher incidence of complex karyotypes (16.3% vs. 3.7%, P = 0.031). No significant differences were noted in 3-year overall survival (OS) (64.3% vs. 71.9%, P = 0.468), leukemia-free survival (LFS) (57.6% vs. 70.8%, P = 0.218), graft-versus-host disease/relapse-free survival (GRFS) (43.3% vs. 53.0%, P = 0.359) and NRM (23.9% vs. 11.7%, P = 0.246). In an exploratory landmark analysis, the solid tumor cohort appeared to have significantly lower OS (P = 0.030), LFS (P = 0.009), and GRFS (P = 0.038) from 2 years after transplantation. Multivariable analysis identified age greater than 55 years, baseline platelet counts less than 50×10^9^/L as significant predictors of inferior OS and LFS in solid tumor patients.

**Conclusion:**

Patients with hematologic diseases secondary to solid tumors showed no significant increase in overall transplantation risk. However, their adverse clinical characteristics and reduced long-term survival rates beyond 2 years post-transplantation, underscore the need to refine HCT-CI scoring and improve management strategies.

## 1 Introduction

Therapy-related hematologic neoplasms (t-HNs) are secondary hematologic malignancies that emerge following cytotoxic chemotherapy or radiotherapy administered for prior solid tumors or hematologic malignancies. This category primarily encompasses myeloid neoplasms post cytotoxic therapy (MN-PCT) (1), including therapy-related myelodysplastic syndrome/acute myeloid leukemia (t-MDS/t-AML) (2) and therapy-related myelodysplastic/myeloproliferative neoplasms (t-MDS/MPN), as well as therapy-related acute lymphoblastic leukemia (t-ALL) (3). With the rising incidence of solid tumors, t-MDS/t-AML constitutes approximately 10-20% of all MDS/AML cases (4), whereas t-ALL accounts for roughly 10% of ALL cases (5). The development of t-HNs results from the interplay of aging, chronic inflammation, genetic susceptibility, and clonal selection, leading to complex cytogenetic abnormalities, high-risk molecular profiles, and generally poor clinical outcomes. Collectively, these characteristics define t-HNs as a distinct biological and clinical entity (6, 7). Compared with those arising from hematologic malignancies, t-HNs secondary to solid tumors exhibit greater heterogeneity and aggressiveness. However, research focusing on this distinction remains limited, potentially obscuring their unique characteristics (8). Despite challenges such as poor performance status and reduced tolerance to chemotherapy in t-HNs patients, hematopoietic stem cell transplantation (HSCT) remains a recommended curative option.

The Hematopoietic Cell Transplantation-Specific Comorbidity Index (HCT-CI) serves as a standardized instrument for evaluating the burden of comorbidities prior to HSCT. It is instrumental in predicting non-relapse mortality (NRM) to guide treatment decisions (9). Among the 17 comorbidities included in the HCT-CI, a score greater than three is significantly correlated with an increased risk of NRM and poorer overall survival (OS) (10). A history of solid malignancy is assigned three points, categorizing it as high risk. Nonetheless, the independent effect of prior solid tumors on transplant outcomes remains controversial (9). Andrew Jay et al. (11) reported no significant differences in OS or relapse rates post-HSCT, regardless of prior solid tumor history. Conversely, other research indicated that either prior solid tumors or hematologic malignancies elevated NRM (8). Given the substantial weight attributed to prior solid tumor history in the HCT-CI, it is imperative to assess its influence on transplant outcomes to refine prognostic assessment and improve clinical management. In this study, we utilized a propensity score matching (PSM) model to retrospectively examine the differences in clinical characteristics, genetic profiles, and transplant outcomes between patients with a history of solid tumors and those with *de novo* hematologic malignancies.

## 2 Materials and methods

### 2.1 Study population and design

We retrospectively analyzed 3,725 consecutive patients who underwent either autologous or allogeneic HSCT at the Institute of Hematology & Blood Diseases Hospital, Chinese Academy of Medical Sciences between January 1, 2014, and July 1, 2024. Inclusion criteria were: (1) primary diagnosis of AML, ALL, or MDS; (2) age ≥18 years at HSCT; (3) first autologous or allogeneic HSCT at our center; and (4) availability of complete clinical data.

**Figure 1:**
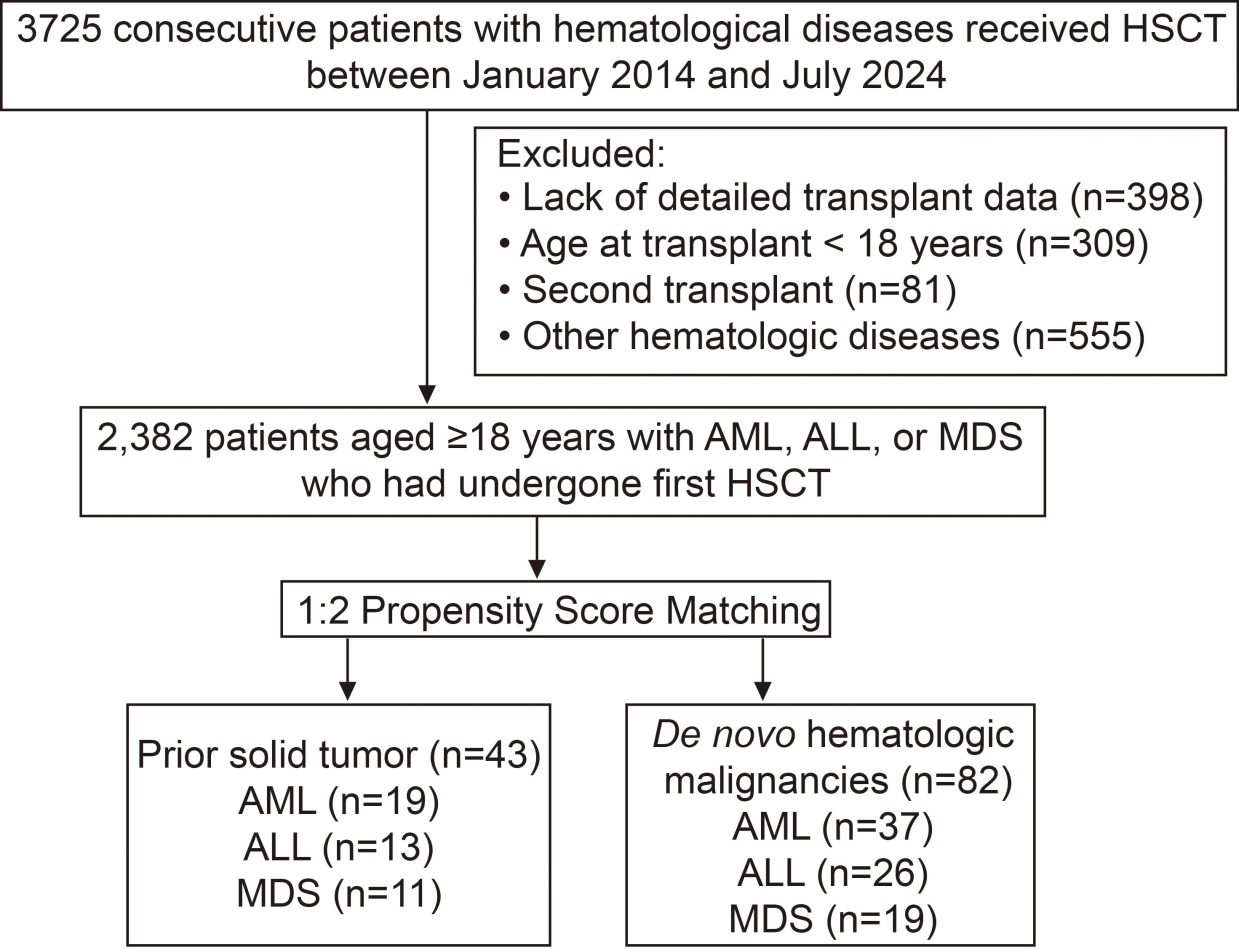
Flow chart presenting inclusion and exclusion criteria of patients enrolled in the study.

Ultimately, 2,382 patients (63.9%) met the inclusion criteria, including 43 patients (1.8%) with a history of malignant solid tumors. To minimize potential confounding, we employed a 1:2 PSM method to match these patients with those diagnosed with *de novo* hematologic malignancies. Previous studies (6, 7) have shown that patients with t-HNs are markedly enriched for adverse cytogenetic and molecular abnormalities, which may have a profound impact on prognosis. To faithfully reflect the biological differences between the two groups, our PSM model was therefore constructed using only clinical and transplant-related covariates, without incorporating disease-specific genomic risk stratification. Matching variables included: (1) patient factors: hematologic diagnosis, age (≤44, 45-59, ≥60 years), sex, and disease status (acute leukemia: CR1, CR2, PR/NR; for MDS: untreated or chemotherapy/demethylating therapy); (2) transplant factors: transplant year (2014-2019, 2020-2024), transplant type (sibling-matched, unrelated-matched, haploidentical, autologous); (3) donor factors: sex, age (<30, 30-45,>45 years) (**Figure 1**). All participants provided written informed consent for use of their clinical data. The study was conducted in accordance with the Declaration of Helsinki and approved by the institutional ethics committee.

### 2.2 Transplant procedures

Conditioning regimens were selected according to the patient’s age, primary diagnosis, and pre-transplant condition, comprising high-dose myeloablative conditioning (MAC, n=119) and reduced-intensity conditioning (RIC, n=6) at our center (12). RIC regimens were defined by meeting the following criteria: (1) single-dose total body irradiation (TBI) ≤ 5 Gy or a cumulative dose ≤ 8 Gy; (2) busulfan dose < 9 mg/kg; (3) melphalan dose < 140 mg/m²; and (4) thiotepa dose ≤ 10 mg/kg. Regimens exceeding these thresholds were classified as MAC. For graft-versus-host disease (GVHD) prophylaxis, patients generally received tacrolimus or cyclosporine in combination with short-course methotrexate and mycophenolate mofetil (13).

### 2.3 Outcomes and definitions

The primary endpoint was OS. Secondary endpoints included leukemia-free survival (LFS), NRM, GVHD-free/relapse-free survival (GRFS), and the cumulative incidence of relapse (CIR). OS was defined from HSCT to death from any cause or the last follow-up and LFS as the time from HSCT to relapse or death. NRM referred to death from causes other than relapse during disease remission. GRFS was defined as survival without grade III-IV acute GVHD (aGVHD), chronic GVHD (cGVHD) requiring systemic treatment, disease relapse, or death. Relapse was characterized by morphological evidence of disease in peripheral blood, bone marrow, extramedullary sites, or by the reappearance of pre-transplant chromosomal abnormalities. All time-to-event endpoints were measured from the date of HSCT to the event of interest and were censored at the last follow-up. MRD positivity was defined as the detection of any measurable disease by multiparameter flow cytometry. Neutrophil engraftment was defined as the first of three consecutive days with an absolute neutrophil count ≥ 0.5×10^9^/L, while platelet engraftment was defined as the first of seven consecutive days with a platelet count ≥ 20×10^9^/L without transfusion support. aGVHD and cGVHD were graded according to MAGIC criteria (14) and NIH consensus guidelines (15). To ensure comparability of disease risk, patients’ primary diseases were stratified using the Disease Risk Index (DRI) model (16). The intensity of the conditioning regimen was evaluated using the Transplant Conditioning Intensity (TCI) score (17). Risk stratification for AML and MDS was conducted using the 2022 European LeukemiaNet (ELN) criteria (18) and the International Prognostic Scoring System (IPSS) (19). The risk classification for ALL was based on the 2024 National Comprehensive Cancer Network (NCCN) guidelines (20).

### 2.4 Statistical analysis

Comparisons between groups were performed using independent t-tests or Wilcoxon rank-sum tests for continuous variables, and chi-square or Fisher’s exact tests for categorical variables. Univariate analyses were performed using Cox proportional hazards regression models, whereas multivariable analyses were conducted using Firth’s penalized Cox regression due to the limited number of events. OS, LFS, and GRFS were estimated using the Kaplan-Meier method, and median follow-up was calculated using the reverse Kaplan-Meier approach by log-rank test. The cumulative incidences of NRM, CIR, grade 3–4 aGVHD within 100 days and cGVHD were estimated using the Fine-Gray subdistribution hazard model, with deaths without the event of interest treated as competing risks. A two-sided P-value <0.05 was considered statistically significant. PSM was conducted at a 1:2 ratio using a caliper width of 0.02. Descriptive and regression analyses were performed using SPSS v27.0 (SPSS Inc., Chicago, IL, USA), whereas PSM, competing risk analyses, landmark analyses and Firth’s penalized Cox regression were conducted in R v4.4.3.

## 3 Results

### 3.1 Clinical characteristics of prior solid tumors

Breast cancer was the most prevalent solid tumor (21/43, 48.8%), followed by gynecologic tumors (6/43, 14.0%, including cervical and ovarian), gastrointestinal tumors (4/43, 9.3%), thyroid cancer (3/43, 7.0%), and lung, brain, and urinary tract tumors (2/43 each, 4.7%). Most patients were female (30/43, 69.8%), with a median age of 45 years (IQR 40-50) at solid tumor diagnosis. Nearly all underwent surgery (41/43, 95.3%), chemotherapy (34/43, 79.1%), radiotherapy (20/43, 46.5%), or chemotherapy/radiotherapy (36/43, 83.7%; collectively referred to as cytotoxic therapy). Those who received cytotoxic therapy met the classical criteria for t-HNs, whereas the 16.3% treated with surgery alone were classified as non–therapy-related hematologic malignancies arising after a prior solid tumor. Tumor control was generally favorable, with 81.4% (35/43) of cases remaining stable, 9.3% (4/43) experiencing prior relapse, and 9.3% (4/43) showing prior metastasis, all of which were stable before HSCT. Secondary hematologic malignancies emerged at a median age of 51.0 years (IQR: 40-50), with 53.5% (23/43) occurring 1–5 years post-diagnosis. These cases comprised AML (19/43, 44.2%), ALL (13/43, 30.2%), and MDS (11/43, 25.6%). Detailed clinical characteristics are provided in **Supplementary Table 1**.

### 3.2 Comparison of characteristics between patients with prior solid tumors and *de novo* hematologic diseases

This study enrolled 2,382 patients, including 2,339 (98.2%) with *de novo* hematologic diseases and 43 (1.8%) with prior solid tumors. Before PSM (**Table 1**), significant differences were noted in transplant age (P<0.001), sex (P = 0.001), and transplant year (P = 0.010), with the solid tumor group being older, having a higher female proportion (69.8% vs. 43.8%), and undergoing HSCT after 2019 (86.0% vs. 66.2%). After PSM adjustment, one solid tumor patient remained unmatched, and two were paired with a single control, resulting in a final cohort of 125 patients (43 in the solid tumor group and 82 in the control group), all receiving peripheral blood-derived stem cells. Compared to the *de novo* group (**Table 2**), the solid tumor group exhibited higher baseline ECOG scores (≥ 2: 23.1% vs. 9.5%, P = 0.049) and HCT-CI (≥ 3: 100% vs. 2.4%, P<0.001), lower platelet counts (median: 35.5 vs. 72×10^9^/L, P = 0.010), a higher incidence of complex karyotypes (16.3% vs. 3.7%, P = 0.031) at diagnosis, and increased frequencies of *KMT2A* rearrangements (25.6% vs. 11.8%, P = 0.010) and *FLT3* mutations (32.6% vs. 11.0%, P = 0.003). No significant differences were observed in DRI risk stratification, conditioning regimen intensity, or neutrophil and platelet engraftment. We further repeated the cytogenetic and molecular subgroup analyses after excluding the 16.3% (7/43) patients without prior cytotoxic therapy, and the results remained largely unchanged (**Supplementary Table 2**).

**Table 1:** Patient characteristics before and after propensity score matching.

Characteristic	Before PSM				After PSM		
	Prior solid tumor (n=43)	*De novo* hematologic disease (n=2,339)	P	SMD	*De novo* hematologic malignancies (n=82)	P	SMD
Age (years), n (%)*			**<0.001**	0.663		0.910	0.082
≤44	14 (32.6%)	1496 (64.0%)			30 (36.6%)		
45-59	27 (62.8%)	793 (33.9%)			49 (59.8%)		
≥60	2 (4.7%)	50 (2.1%)			3 (3.7%)		
Sex, n (%)*			**0.001**	0.544		1.000	0.010
Male	13 (30.2%)	1315 (56.2%)			25 (30.5%)		
Female	30 (69.8%)	1024 (43.8%)			57 (69.5%)		
**Diagnosis, n (%)**			0.859	0.085		0.995	0.019
AML	19 (44.2%)	1132 (48.4%)			37 (45.1%)		
ALL	13 (30.2%)	647 (27.7%)			26 (31.7%)		
MDS	11 (25.6%)	560 (23.9%)			19 (23.2%)		
**Disease status, n (%)**			0.923	0.168		0.987	0.110
CR1	27 (62.8%)	1463 (62.5%)			54 (65.9%)		
CR2 or higher	1 (2.3%)	126 (5.4%)			3 (3.7%)		
PR/NR	4 (9.3%)	190 (8.1%)			6 (7.3%)		
Untreated	7 (16.3%)	338 (14.5%)			11 (13.4%)		
Chemotherapy/ Demethylating therapy	4 (9.3%)	222 (9.5%)			8 (9.8%)		
Transplant year, n (%)*			**0.010**	0.478		1.000	0.025
2014-2018	6 (14.0%)	790 (33.8%)			11 (13.4%)		
2019-2024	37 (86.0%)	1549 (66.2%)			71 (86.6%)		
**Transplant type, n (%)**			0.785	0.228		0.945	0.119
Sibling-matched	13 (30.2%)	752 (32.2%)			23 (28.0%)		
Unrelated-matched	1 (2.3%)	100(4.3%)			3 (3.7%)		
Haploidentical	24 (55.8%)	1278 (54.6%)			44 (53.7%)		
Autologous	5 (11.6%)	182 (7.8%)			12 (14.6%)		
Unrelated-mismatched	0 (0%)	27 (1.2%)			0 (0%)		
**Donor sex, n (%)**			0.120	0.280		1.000	0.015
Male	33 (76.7%)	1499 (64.1%)			63 (76.8%)		
Female	10 (23.3%)	840 (35.9%)			19 (23.2%)		
**Donor age (years), n (%)**			0.343	0.233		0.876	0.098
<30	11 (25.6%)	838 (35.8%)			21 (25.6%)		
30-45	20 (46.5%)	882 (37.7%)			36 (43.9%)		
>45	12 (27.9%)	619 (26.5%)			25 (30.5%)		

AML, acute myeloid leukemia; ALL, acute lymphoblastic leukemia; MDS, myelodysplastic syndromes; CR1, first complete remission; CR2, second complete remission; PR, partial remission; NR, no response.

The boldface formatting applied to values is intended to highlight statistically significant results (defined as p<0.05).

*indicates statistical significance (P< 0.05).

**Table 2:** Comparison of transplant characteristics between patients with prior solid tumors and *de novo* hematologic malignancies. .

Characteristic	Total (n=125)	Prior solid tumor (n=43)	*De novo* hematologic malignancies (n=82)	P
**Age at transplantation, years, median (IQR)**	48 (40.0, 53.0)	51 (40.0, 55.0)	47 (39.8, 52.0)	0.181
**ECOG at diagnosis, n (%)**				**0.049**
0-1	97 (85.8%)	30 (76.9%)	67 (90.5%)	
≥2	16 (14.2%)	9 (23.1%)	7 (9.5%)	
Missing	12	4	8	
**ECOG at transplant, n (%)**				0.920
0-1	99 (87.6%)	34 (87.2%)	65 (87.8%)	
≥2	14 (12.4%)	5 (12.8%)	9 (12.2%)	
Missing	12	4	8	
**HCT-CI**				<0.001
0 (Low Risk)	47 (37.6%)	0 (0%)	47 (57.3%)	
1-2 (Intermediate Risk)	33 (26.4%)	0 (0%)	33 (40.2%)	
≥3 (High Risk)	45 (36.0%)	43 (100%)	2 (2.4%)	
**Hematologic disease at diagnosis**				
WBC, ×10^9^/L, median (IQR)	5.2 (2.6, 22.8)	6.9 (2.9, 27.7)	4.7 (2.6, 18.7)	0.387
PLT, ×10^9^/L, median (IQR)	58.5 (29.8, 115.3)	35.5 (22.3, 84.8)	72.0 (35.5, 128.5)	**0.010**
HGB, g/L, median (IQR)	83.5 (68, 101.3)	87.0 (73.3, 107.8)	82.5 (67.5, 96.0)	0.418
Bone marrow blasts (%)	49.0 (14.0, 83.0)	49.0 (14.5, 84.0)	50.3 (12.1, 80.0)	0.598
**Complex karyotype, n (%)**				**0.031**
Yes	10 (8.0%)	7 (16.3%)	3 (3.7%)	
No	115 (92.0%)	36 (83.7%)	79 (96.3%)	
**DRI risk stratification, n (%)**				0.196
Low + Intermediate risk	106 (84.8%)	34 (79.1%)	72 (87.8%)	
High risk	19 (15.2%)	9 (20.9%)	10 (12.2%)	
**Molecular Biology**				
*KMT2A* rearrangement, n (%)	18 (14.4%)	11 (25.6%)	7 (11.8%)	**0.010**
*TP53* mutation, n (%)	7 (5.6%)	4 (9.3%)	3 (3.7%)	0.231
*FLT3* mutation, n (%)	23 (18.4%)	14 (32.6%)	9 (11.0%)	**0.003**
*DNMT3A* mutation, n (%)	13 (10.4%)	3 (7.0%)	10 (12.2%)	0.540
*RUNX1* mutation, n (%)	13 (10.4%)	6 (14.0%)	7 (8.5%)	0.367
**Conditioning regimen intensity, n (%)**				0.661
MAC	119 (95.2%)	41 (95.3%)	78 (95.1%)	
RIC	6 (4.8%)	2 (4.7%)	4 (4.9%)	
**Transplant conditioning intensity, n(%)**				0.187
1-3.5	31 (39.7%)	8 (29.6%)	23 (45.1%)	
4-6	47 (60.3%)	19 (70.4%)	28 (30.7%)	
**Time from diagnosis to transplant, months, median (IQR)**	7.0 (5.0, 9.0)	7.0 (5.0, 8.0)	7.0 (5.0, 9.0)	0.275
Pre-transplant MRD, n (%)*				0.762
Negative	73 (76.8%)	24 (75.0%)	49 (77.8%)	
Positive	22 (23.2%)	8 (25.0%)	14 (22.2%)	
**MNC, ×10^8^/kg, median (IQR)**	10.0 (8.0, 12.0)	10.2 (8.0, 12.2)	10.0 (8.0, 12.0)	0.456
**CD34^+^ cell count, ×10^6^/kg, median (IQR)**	3.3 (2.6, 4.5)	3.4 (2.8, 4.2)	3.2 (2.5, 4.9)	0.662
**Neutrophil engraftment, days, median (IQR)**	13.0 (11.0, 15.0)	13.0 (11.0, 15.0)	13.0 (11.0, 15.0)	0.673
**Platelet engraftment, days, median (IQR)**	15.0 (13.0, 24.5)	15.0 (13.0, 24.0)	13.0 (13.0, 25.0)	0.827

ECOG, eastern cooperative oncology group; HCT-CI, hematopoietic cell transplantation-specific comorbidity index; WBC, white blood cell; PLT, platelet; HGB, hemoglobin; DRI, disease risk index; MAC, myeloablative conditioning; RIC, reduced-intensity conditioning; MRD, minimal residual disease (*assessed only for acute leukemia); MNC, mononuclear cell.

The boldface formatting applied to values is intended to highlight statistically significant results (defined as p<0.05).

### 3.3 Transplant outcomes between patients with prior solid tumors and *de novo* hematologic diseases

**Figure 2:**
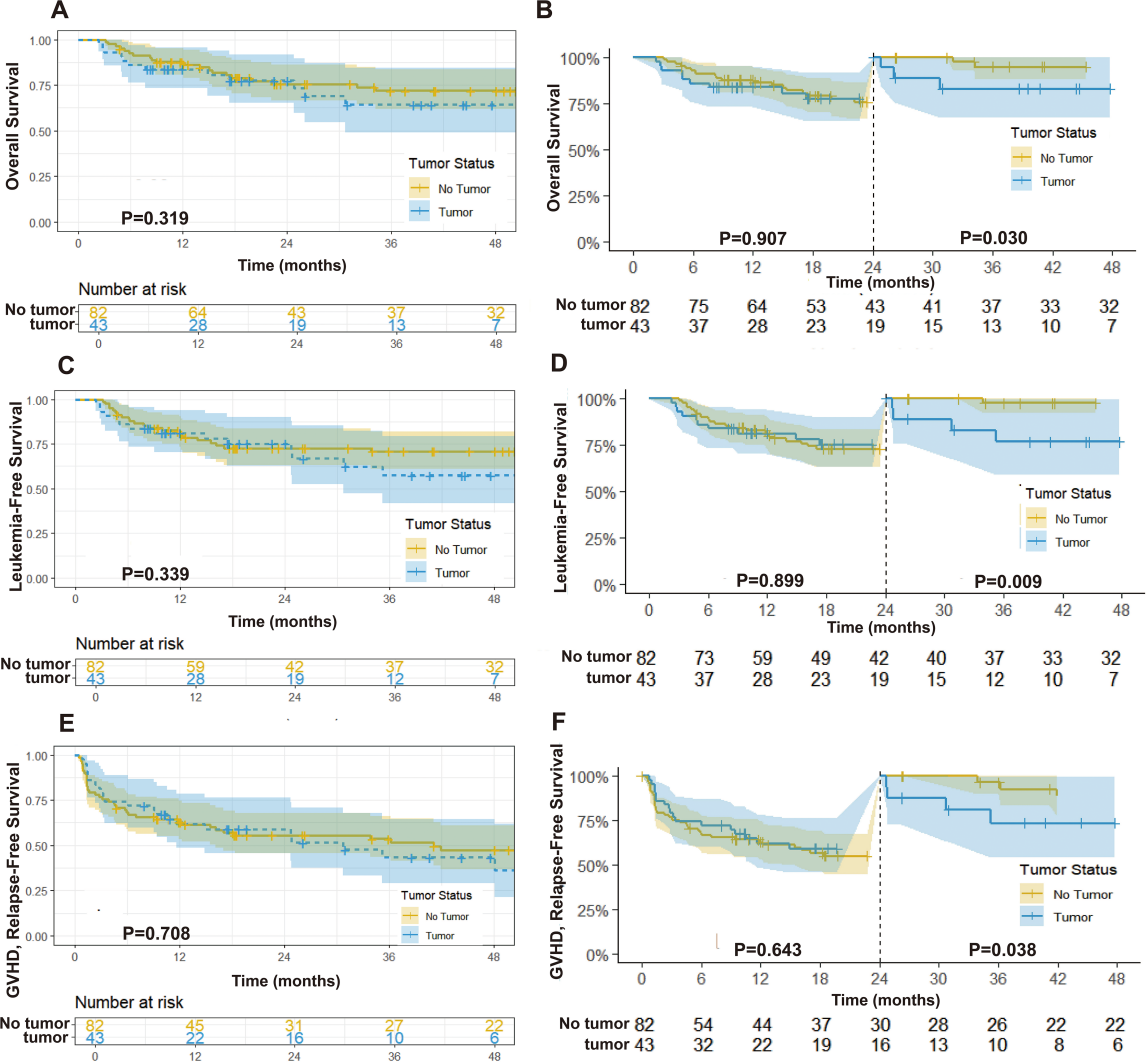
Kaplan-Meier curves for overall survival **(A)**, leukemia-free survival **(C)**, and GVHD-free/relapse-free survival **(E)**. Landmark curves for overall survival **(B)**, leukemia-free survival **(D)**, and GVHD-free/relapse-free survival **(F)**.

**Figure 3:**
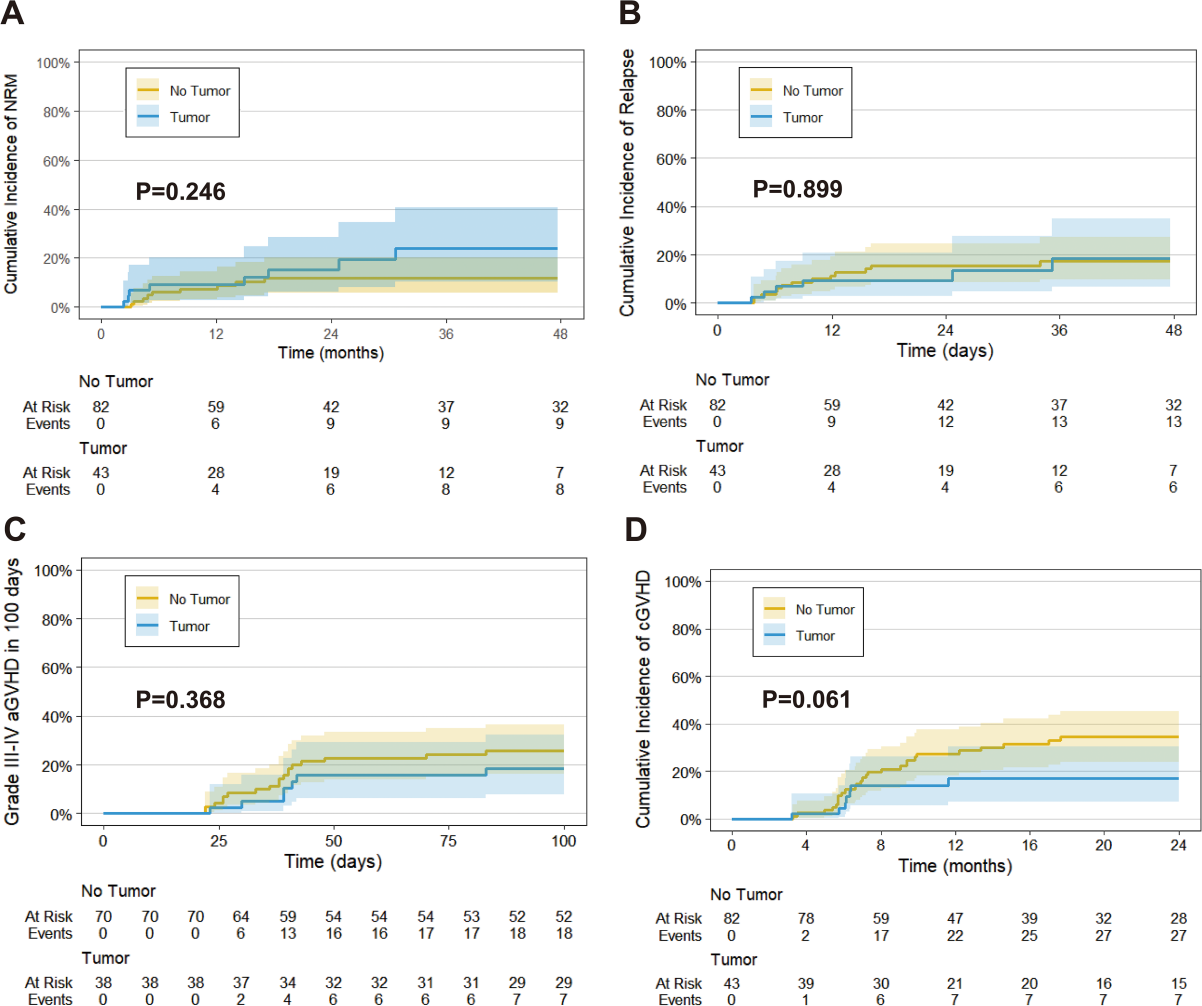
Non-relapse mortality **(A)**. Cumulative incidence of relapse **(B)**. Cumulative incidence of III-IV aGVHD in 100 days **(C)**. Cumulative incidence of cGVHD within 2 years **(D)**.

The median follow-up time was 31.0 months (IQR: 10.9-51.1) for the prior solid tumor group and 49.8 months (IQR: 39.9-59.7) for the *de novo* hematologic disease group, with no significant difference (P = 0.155). During follow-up, only one patient in the prior solid tumor group experienced primary tumor relapse and metastasis (lung cancer with brain metastasis). Mortality rates were 30.2% (13/43) in the prior solid tumor group, attributed to relapse (4 cases), infection (6 cases), aGVHD (2 cases), and heart failure (1 case), compared to 24.4% (20/82) in the *de novo* group, due to relapse (11 cases), infection (5 cases), aGVHD (3 cases), and heart failure (1 case). No significant differences were found between groups in 3-year OS (64.3% [95% CI: 49.0%-84.4%] vs. 71.9% [95% CI: 61.9%-83.5%], P = 0.468), LFS (57.6% [95% CI: 41.8%-79.2%] vs. 70.8% [95% CI: 61.1%-82.1%], P = 0.218), GRFS (43.3% [95% CI: 29.0%-64.6%] vs. 53.0% [95% CI: 43.4%-66.3%], P = 0.359) (**Figure 2**). Similarly, there were no significant differences in NRM (23.9% [95% CI: 10.5%-40.5%] vs. 11.7% [95% CI: 5.7%-20.0%], P = 0.246), or CIR (18.5% [95% CI: 6.7%-34.8%] vs. 17.5% [95% CI: 9.7%-27.1%], P = 0.899) (**Figure 3**). Adjusted subgroup analyses (controlling for age, sex, disease type, baseline white blood cell and platelet counts, bone marrow blasts, DRI category, MRD status, and transplant type) showed no significant differences in OS or LFS between the two groups (**Supplementary Figure 2**). In a supplementary exploratory 2-year landmark analysis, we observed separation of the survival curves beyond 2 years, with patients with prior solid tumors showing shorter OS (P = 0.03), LFS (P = 0.009), and GRFS (P = 0.038) from that time point onward (**Figure 2**).

No statistically significant differences were observed in the cumulative incidences of grade 3–4 aGVHD within 100 days (18.4% [95% CI: 8.0%–32.2%] vs. 25.7% [95% CI: 16.1%–36.4%], P = 0.368) (**Figure 3C**), overall cGVHD within 2 years (17.1% [95% CI: 7.3%–30.4%] vs. 34.4% [95% CI: 24.0%–45.0%], P = 0.061) (**Figure 3D**), or moderate-to-severe cGVHD (12.4% [95% CI: 4.4%–24.8%] vs. 18.4% [95% CI: 10.5%–28.0%], P = 0.447) (**Table 3**).3.4 Prognostic factors for transplant outcomes in patients with prior solid tumors.

**Table 3:** The results of the matched-pair analysis between patients with prior solid tumors and *de novo* hematologic malignancies.

Outcome	Prior solid tumor (n=43)	*De novo* hematologic malignancies (n=82)	P
3-year OS	64.3% [95% CI: 49.0%-84.4%]	71.9% [95% CI: 61.9%-83.5%]	0.468
3-year LFS	57.6% [95% CI: 41.8%-79.2%]	70.8% [95% CI: 61.1%-82.1%]	0.218
3-year GRFS	43.3% [95% CI: 29.0%-64.6%]	53.0% [95% CI: 43.4%-66.3%]	0.359
3-year NRM	23.9% [95% CI: 10.5%-40.5%]	11.7% [95% CI: 5.7%-20.0%]	0.246
3-year CIR	8.5% [95% CI: 6.7%-34.8%]	17.5% [95% CI: 9.7%-27.1%]	0.899
grade 3–4 aGVHD within 100 days	18.4% [95% CI: 8.0%–32.2%]	25.7% [95% CI: 16.1%–36.4%]	0.368
cGVHD within 2 years	17.1% [95% CI: 7.3%–30.4%]	34.4% [95% CI: 24.0%–45.0%]	0.061
moderate-to-severe cGVHD within 2 years	12.4% [95% CI: 4.4%–24.8%]	18.4% [95% CI: 10.5%–28.0%]	0.447

OS, Overall Survival; LFS, Leukemia-Free Survival; GRFS, Graft versus Host Disease-free/relapse-free survival; NRM, Non-Relapse Mortality; aGVHD, Acute Graft-versus-Host Disease; cGVHD, Chronic Graft-versus-Host Disease.

Univariate Cox regression analysis identified key prognostic factors for OS and LFS in patients with prior solid tumors (**Table 4**). Age >55 years at HSCT (OS: HR = 3.69, 95% CI: 1.16-11.74, P = 0.027; LFS: HR = 4.56, 95% CI: 1.49-13.93, P = 0.008), complex karyotype (OS: HR = 3.52, 95% CI: 1.05-11.74, P = 0.041; LFS: HR = 3.58, 95% CI: 1.19-10.74, P = 0.023) and *TP53* mutation (OS: HR = 6.31, 95% CI: 1.67-23.85, P = 0.007; LFS: HR = 4.90, 95% CI: 1.34-17.86, P = 0.016) were significantly associated with worse outcomes for both OS and LFS. A platelet count ≤ 50×10^9^/L at diagnosis emerged as an adverse factor for LFS (HR = 5.23, 95% CI: 1.17-23.37, P = 0.030).

**Table 4:** Univariate analysis of prognostic factors in patients with prior solid tumors.

Characteristic	OS		LFS	
	HR (95% CI)	P	HR (95% CI)	P
**ECOG change**		0.059		0.181
Unchanged or improved	Reference		Reference	
Worsened	3.02 (0.96-9.55)		2.11 (0.71-6.32)	
**Age at transplant (years)**		**0.027**		**0.008**
≤ 55	Reference		Reference	
>55	3.69 (1.16-11.74)		4.56 (1.49-13.93)	
**Breast cancer**		0.595		0.236
No	Reference		Reference	
Yes	0.74 (0.25-2.22)		0.53 (0.19-1.51)	
**Time from tumor diagnosis to secondary hematologic disease (years)**		0.465		0.267
≤5	Reference		Reference	
>5	1.49 (0.51-4.35)		1.78 (0.64-4.98)	
**Received chemotherapy**		0.381		0.798
No	Reference		Reference	
Yes	2.27 (0.29-17.73)		1.23 (0.24-5.53)	
**Received radiotherapy**		0.118		0.618
No	Reference		Reference	
Yes	0.40 (0.12-1.32)		0.62 (0.22-1.75)	
**Received combined chemo/radiotherapy**		0.198		0.247
No	Reference		Reference	
Yes	0.46 (0.14-1.50)		0.52 (0.18-1.56)	
**Received cytotoxic therapy(radiotherapy or chemotherapy)**		0.607		0.495
No	Reference		Reference	
Yes	0.58 (0.075-4.54)		0.49 (0.064-3.77)	
**Prior relapse or metastasis**		0.592		0.143
No	Reference		Reference	
Yes	1.45 (0.39-19.12)		2.37 (0.79-7.09)	
**Platelet count at diagnosis (×10^9^/L)**		**0.063**		**0.030**
≤50	4.21 (0.93-1.00)		5.23 (1.17-23.37)	
>50	Reference		Reference	
**Complex karyotype**		**0.041**		**0.023**
No	Reference		Reference	
Yes	3.52 (1.05-11.74)		3.58 (1.19-10.74)	
**DRI score**		**0.053**		0.139
Low or intermediate risk	Reference		Reference	
High risk	3.11 (0.98-9.81)		2.29 (0.76-6.84)	
***KMT2A* rearrangement**				
No	Reference	0.591	Reference	0.937
Yes	0.66 (0.14-3.03)		0.95 (0.26-3.45)	
***TP53* mutation**				
No	Reference	**0.007**	Reference	**0.016**
Yes	6.31 (1.67-23.85)		4.90 (1.34-17.86)	
***FLT3* mutation**				
No	Reference	0.495	Reference	0.300
Yes	0.63 (0.17-2.35)		0.51 (0.14-1.82)	
***RUNX1* mutation**				
No	Reference	0.497	Reference	0.401
Yes	0.50 (0.063-3.813)		0.42 (0.054-3.20)	

The boldface formatting applied to values is intended to highlight statistically significant results (defined as p<0.05).

Multivariable Firth-penalized Cox regression identified age > 55 years (OS: HR = 6.75, 95% CI: 1.71-26.7, P = 0.007; LFS: HR = 5.56, 95% CI: 1.51-20.50, P = 0.012), baseline platelet counts ≤ 50×10^9^/L (OS: HR = 3.83, 95% CI: 0.86-16.98, P = 0.041; LFS: HR = 4.79, 95% CI: 1.12-20.50, P = 0.012) as significant predictors of inferior OS and LFS in solid tumor patients (**Supplementary Table 3**).

## 4 Discussion

Despite growing interest in the treatment strategies and prognostic factors of t-HNs (21), there is still a paucity of evidence regarding the clinical features and post-transplant outcomes of patients with a history of solid tumors. In our cohort, the solid tumors group presented with inferior ECOG performance status, which may reflect residual effects of prolonged exposure to cytotoxic therapy and associated physiological exhaustion. They also showed significantly lower baseline platelet counts and a higher frequency of complex karyotypes. Prior exposure to alkylating agents, topoisomerase II inhibitors, or radiotherapy can impair the bone marrow microenvironment, particularly megakaryocytes, reducing platelet production (22). HSCT may reset this microenvironment (23), resulting in comparable platelet engraftment between groups. Frequent cytotoxic chemotherapy and radiotherapy also induce DNA damage and repair-pathway defects (24). We observed higher frequencies of *KMT2A* rearrangements and complex karyotype in t-HNs group. Previous studies have shown that exposure to topoisomerase II inhibitors is associated with an increased incidence of *KMT2A* rearrangements, whereas treatment with alkylating agents tends to induce complex or monosomal karyotypic abnormalities (5, 25, 26). These drug-related mechanisms of genomic and chromosomal damage could partially explain the higher prevalence of adverse molecular events observed in t-HNs. However, No significant differences were observed in conditioning regimens, or time from diagnosis to transplant, which contrasted with some reports (11), suggesting that regimen decisions at our center might prioritize primary disease malignancy and pre-transplant status over solid tumor history. Furthermore, the cumulative incidences of grade 3–4 aGVHD, moderate-to-severe cGVHD, and CIR were similar between the two groups. This may reflect the predominantly MAC-based regimens, broadly comparable donor matching, and largely uniform GVHD prophylaxis. Univariate analysis identified several predictors of poorer OS and LFS, including age > 55 years, platelet count ≤ 50×10^9^/L, complex karyotype, high-risk DRI, and *TP53* mutation. Subsequent multivariable analysis using the Firth-penalized Cox model confirmed that older age and lower platelet count remained significant independent adverse factors, consistent with established risk factors for adverse transplant outcomes. Aligned with our findings, a study of 565 Japanese patients with t-HNs also demonstrated that advanced age, delayed transplantation, poor performance status, and unfavorable cytogenetics were independent predictors of inferior post-transplant survival (27). Peking University People’s Hospital conducted a nomogram analysis of 154 s-AML patients who underwent HSCT. The model stratified risk based on initial white blood cell count, genetic profile, delayed platelet engraftment, age at solid tumor diagnosis, and relapse status to improve prognostic prediction (28). Although early studies of Magrolimab (29) and Sabatolimab (30) have shown encouraging results, the management of t-HMs harboring *TP53* mutations and complex karyotypes remains challenging (31). *TP53* mutations was associated with a 6.31-fold higher risk of OS, indicating a highly aggressive disease phenotype. Despite a higher incidence of *KMT2A* rearrangement in the solid tumor group, HSCT appeared to partially mitigate their adverse prognostic impact (26). In contrast, solid tumor-specific factors, including breast cancer, a shorter latency period, or prior treatment, did not significantly affect survival. Although shorter latency typically indicated aggressive disease and worse prognosis, this was not observed in our study, which possibly due to a limited sample size or effective pre-transplant disease control, with most patients achieving CR and negative MRD (11, 32). This suggested that transplant outcomes are primarily influenced by the biology of hematologic diseases and the status of the hematopoietic system rather than history of solid tumors.

Accumulating evidence indicates that HSCT significantly improves survival compared with conventional chemotherapy in t-HNs (33, 34). However, the impact of a preceding solid tumor history on transplant outcomes remains uncertain. Compared with the control group, their 3-year OS, LFS, and GRFS were numerically lower, and NRM was numerically higher; however, these differences did not reach statistical significance in the context of limited statistical power. Landmark analysis revealed significantly shorter OS (P = 0.03), LFS (P = 0.009) and GRFS (P = 0.038) in the solid tumor group beyond two years post-transplant. This finding is derived from a small sample with few late events and unequal follow-up between groups, the suggested long-term survival differences should be considered exploratory and hypothesis-generating and interpreted with caution. Despite potential selection bias, overall transplant outcomes for eligible patients with prior solid tumors were comparable when evaluated over the entire follow-up period. Similar outcomes were observed among the subtype secondary to hematologic malignancies (35). For instance, therapy-related chronic myelomonocytic leukemia did not exhibit typical high-risk features or markedly adverse prognosis (36). However, other studies have reported that t-HNs are still associated with higher relapse rates and poorer survival, which is largely attributable to high-risk cytogenetic abnormalities and advanced age (37–39). It was noteworthy that solid tumor group at our center exhibited higher OS and lower NRM compared to other centers. The predominance of breast cancer (48.8%) and its favorable prognosis might partially account for the comparable survival outcomes. A study of 40 patients with secondary hematologic diseases after breast cancer reported a median OS of 1.2 years (range: 0.06-6.5 years), with 12 achieving long-term survival (median: 7.4 years) (40). Patients (n=25) with prior prostate cancer had a median post-transplant OS of 2.5 years (41), underscoring the heterogeneity among solid tumors. Stable control of solid tumors facilitated subsequent intensive treatment of t-HNs. Conversely, a short interval between the two diseases often resulted in poor performance status, precluding timely therapy and leading to inferior outcomes (42). Besides, patients generally showed favorable pre-transplant status, with improved ECOG performance and good disease remission. 84.4% of t-AML patients underwent HSCT during CR1, consistent with a cohort of 351 secondary ALL patients (58.8% secondary to solid tumors), where HSCT in CR1 yielded outcomes comparable to *de novo* ALL (43). It cannot be overlooked that majority of solid tumor patients in our study underwent MAC regimens. The EBMT study on HSCT for t-AML suggested that MAC regimens improved 3-year LFS and OS by 50% compared to RIC, with lower relapse rates and no significant difference in NRM (44). This indicated that the potent immunosuppressive effect of MAC was well suited to these patients. Moreover, although patients had a higher incidence of complex karyotypes and adverse genetic mutations, chemotherapy and targeted therapy pre- and post-transplantation have expanded the therapeutic window (45). These include *FLT3* inhibitors (46), and emerging Menin inhibitors targeting *KMT2A* rearrangements and *NPM1* mutations, which are expected to offer additional treatment opportunities (47). Given the analysis presented, although HCT-CI is widely utilized for pre-transplant risk assessment and effectively predicts OS and NRM, the independent prognostic value of its components necessitates further clarification (48). HCT-CI assigns a uniform score of three for prior solid tumors, potentially overlooking critical factors such as tumor type, control status, and duration of remission. Especially, advancements in tumor therapy since 2005 could lead to an overestimation of transplant risk (9). In our cohort, only one patient experienced relapse of the primary solid tumor within three years. A 10-year follow-up study demonstrated that patients with t-HNs achieved durable survival benefits from HSCT, with a 10-year OS rate of around 24% (49).

Despite potential selection bias and more adverse clinical features at baseline, patients with prior solid tumors who predominantly received MAC conditioning achieved outcomes comparable to those with *de novo* hematologic malignancies. HSCT was recommended for eligible patients with adequate platelet control at diagnosis and non-complex karyotypes. Enhanced management strategies beyond two years post-transplantation are essential for improving long-term survival rates. With rising t-HNs amid increasing tumor incidence, refining HCT-CI to better account for solid tumor heterogeneity is essential for accurate risk prediction.

However, this single-center retrospective study should be interpreted with caution. First, optimal conditioning regimens for t-HNs require validation in larger multicenter cohorts, as our study population was almost uniformly conditioned with MAC, and the very small number of RIC cases precluded any reliable assessment of conditioning intensity. The limited size of the solid tumor cohort markedly reduced statistical power and constrained subgroup, so findings from extended follow-up and the 2-year landmark analysis should be regarded as exploratory and hypothesis-generating. Besides, solid tumors are biologically and clinically heterogeneous, and different tumor types and treatments may differentially affect subsequent transplant outcomes. Our analysis only compared outcomes between breast cancer and other solid tumors, with no significant differences were observed; however, missing data on tumor stage, molecular subtypes, and prior chemotherapy details hindered nuanced tumor-specific impact assessment. Meanwhile, 16.3% patients in the solid tumor cohort had not received prior cytotoxic therapy, and the interpretation of their cytogenetic findings should therefore be made with particular caution. Furthermore, the advent of targeted agents and optimized regimens (50, 51), has transformed the treatment of relapsed or refractory hematologic malignancies. The lack of a chemotherapy control group limited the generalizability of our results. Future studies with larger sample sizes and multi-center prospective designs are needed to further validate the robustness of the conclusions drawn from this study.

## Data Availability

The original contributions presented in the study are included in the article/**Supplementary Material**. Further inquiries can be directed to the corresponding author.
