# Supplementary material for: Impact of prior solid tumor on outcomes of hematopoietic stem cell transplantation for hematologic malignancies: a propensity score-matched study
Source: Front Immunol. 2026 Feb 5;17:1727469. doi: 10.3389/fimmu.2026.1727469 (PMC12916414; doi:10.3389/fimmu.2026.1727469)
Supplement: Supplementary file 1 [file DataSheet1.docx]

Supplementary Material

# Content

**Table**

Supplementary Table 1 Clinical characteristics of patients with Prior malignant solid tumors

Supplementary Table 2 Comparison of transplant characteristics between patients with t-HNs and de novo hematologic malignancies in cytogenetics and molecular biology

Supplementary Table 3 Multivariable Firth-penalized Cox regression analyses of prognostic factors in patients with prior solid tumors

**Figure**

Supplementary Figure 1 Landmark curves for overall survival (A), leukemia-free survival (B), and GVHD-free/relapse-free survival (C)

Supplementary Figure 2 Forest plots of overall survival, leukemia-free survival, and graft-versus-host

# Supplementary Figures and Tables

## Supplementary Table

**Supplementary Table 1** Clinical characteristics of patients with prior malignant solid tumors

| **Features** | n (%) |
| --- | --- |
| **Age at solid tumor diagnosis, years, Median (IQR)** | 45 (35, 51) |
| **Gender** |  |
| Male | 13 (30.2%) |
| Female | 30 (69.8%) |
| **Type of malignant solid tumor** |  |
| Breast cancer | 21 (48.8%) |
| Gynecological tumor | 6 (14%) |
| Gastrointestinal tumor | 4 (9.3%) |
| Thyroid cancer | 3 (7.0%) |
| Lung cancer | 2 (4.7%) |
| Brain cancer | 2 (4.7%) |
| Urological tumor | 2 (4.7%) |
| Other | 3 (7.0%) |
| **Underwent surgery** |  |
| Yes | 41 (95.3%) |
| No | 2 (4.7%) |
| **Underwent chemotherapy** |  |
| Yes | 34 (79.1%) |
| No | 9 (20.9%) |
| **Underwent radiotherapy** |  |
| Yes | 20 (46.5%) |
| No | 23 (53.5%) |
| **Underwent chemotherapy or radiotherapy** |  |
| Yes | 36 (83.7%) |
| No | 7 (16.3%) |
| **Tumor status** |  |
| Stable | 35 (81.4%) |
| Previous relapse | 4 (9.3%) |
| Previous metastasis | 4 (9.3%) |
| **Type of secondary hematologic disease** |  |
| ALL | 13 (30.2%) |
| AML | 19 (44.2%) |
| MDS | 11 (25.6%) |
| **Median age at secondary hematologic disease, years (IQR)** | 51 (40, 55) |
| **Time to secondary hematologic disease, years** |  |
| ≤1 | 7 (16.3%) |
| (1,5] | 23 (53.5%) |
| (5-10] | 9 (20.9%) |
| ＞10 | 4 (9.3%) |

AML, acute myeloid leukemia; ALL: acute lymphoblastic leukemia; MDS: myelodysplastic syndromes.

**Supplementary Table 2** Comparison of transplant characteristics between patients with t-HNs and de novo hematologic malignancies in cytogenetics and molecular biology

|  | **Total (n=125)** | **Prior solid tumor (n=43)** | ***De novo* hematologic malignancies**  **(n=82)** | **P** |
| --- | --- | --- | --- | --- |
| **Complex karyotype, n (%)** |  |  |  | **0.002** |
| Yes | 8 (7.6%) | 7 (19.4%) | 1 (1.4%) |  |
| No | 97 (92.4%) | 29 (80.6%) | 68 (98.6%) |  |
| **DRI risk stratification, n (%)** |  |  |  | 0.077 |
| Low + Intermediate risk | 88 (83.8%) | 27 (75.0%) | 61 (88.4%) |  |
| High risk | 17 (16.2%) | 9 (25.0%) | 8 (11.6%) |  |
| **Molecular Biology** |  |  |  |  |
| *KMT2A* rearrangement, n (%) | 17 (16.2%) | 10 (27.8%) | 7 (10.1%) | **0.020** |
| *TP53* mutation, n (%) | 6 (5.7%) | 3 (8.3%) | 3 (4.3%) | 0.410 |
| *FLT3* mutation, n (%) | 17 (16.2%) | 9 (25.0%) | 8 (11.6%) | 0.077 |
| *DNMT3A* mutation, n (%) | 10 (9.5%) | 1 (2.8%) | 9 (13.0%) | 0.159 |
| *RUNX1* mutation, n (%) | 11 (10.5%) | 5 (13.9%) | 6 (8.7%) | 0.505 |

**Supplementary Table 3** Multivariable Firth-penalized Cox regression analyses of prognostic factors in patients with prior solid tumors

|  | **OS** | | **LFS** | |
| --- | --- | --- | --- | --- |
| **Characteristic** | **HR (95% CI)** | **P** | **HR (95% CI)** | **P** |
| **Age at transplant (years)** |  | **0.007** |  | **0.012** |
| ≤ 55 | Reference |  | Reference |  |
| ＞55 | 6.75 (1.71-26.7) |  | 5.558 (1.51-20.50) |  |
| **Platelet count at diagnosis (×10⁹/L)** |  | **0.041** |  | **0.012** |
| ≤50 | 3.83 (0.86-16.98) |  | 4.79 (1.12-20.5) |  |
| ＞50 | Reference |  | Reference |  |
| **Complex karyotype** |  | 0.847 |  | 0.371 |
| No | Reference |  | Reference |  |
| Yes | 1.17 (0.24-5.63) |  | 1.822 (0.49-6.77) |  |
| **DRI score** |  | 0.182 |  | 0.294 |
| Low or intermediate risk | Reference |  | Reference |  |
| High risk | 2.51 (0.64-9.82) |  | 1.92 (0.56-6.62) |  |

## Supplementary Figure


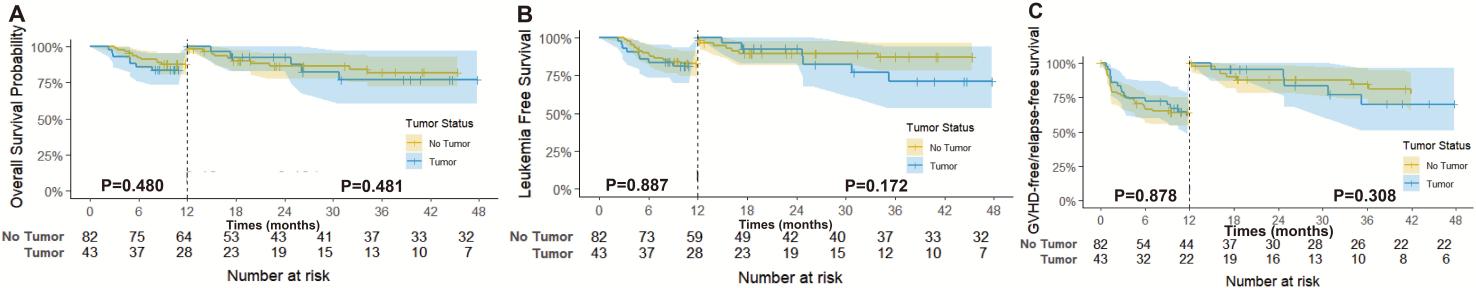


**Supplementary Figure 1** Landmark curves for overall survival (A), leukemia-free survival (B), and GVHD-free/relapse-free survival (C)


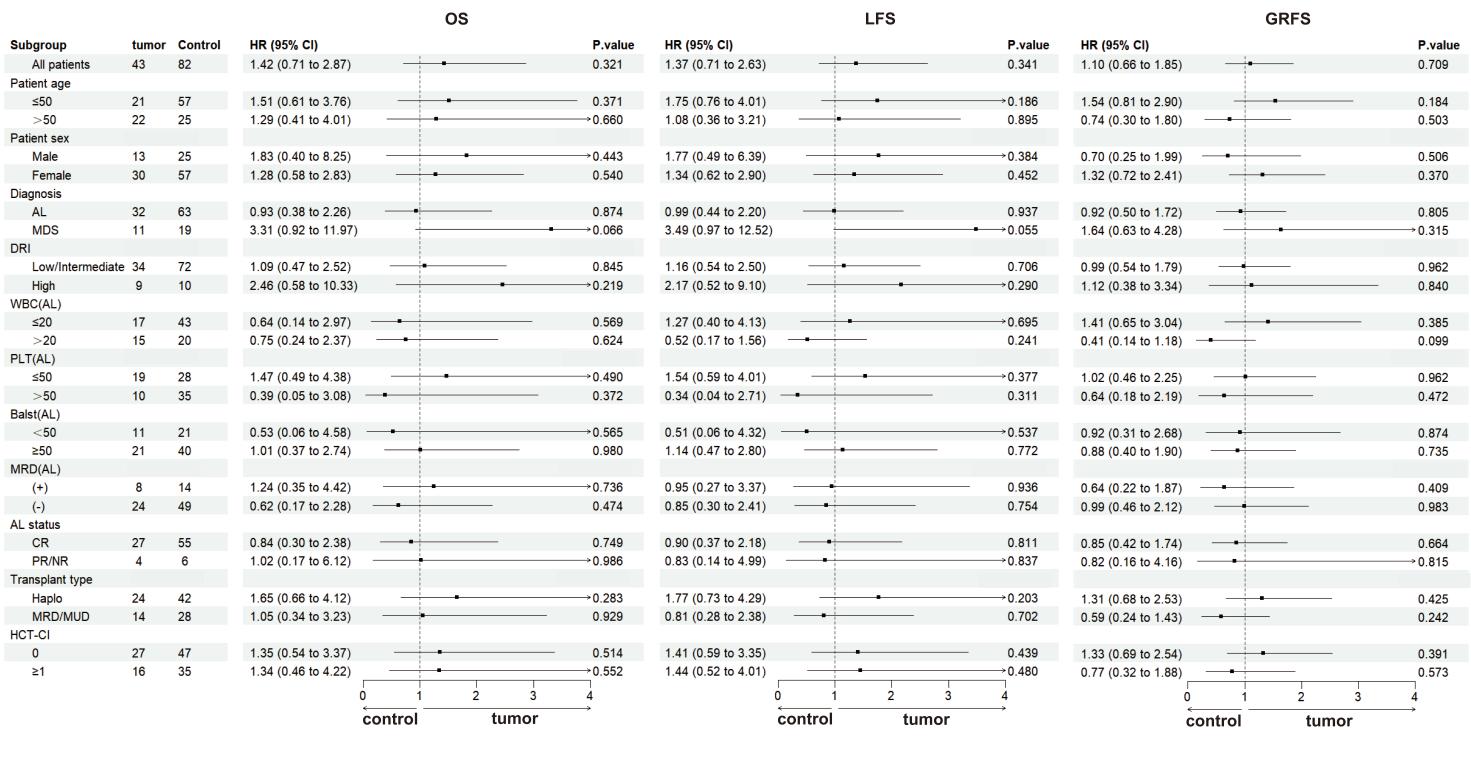


**Supplementary Figure 2** Forest plots of overall survival, leukemia-free survival, and graft-versus-host disease and relapse-free survival in subgroup analyses
